# Morphological and Phylogenetic Analyses Reveal Four New Species of *Gnomoniopsis* (*Gnomoniaceae*, *Diaporthales*) from China

**DOI:** 10.3390/jof8080770

**Published:** 2022-07-25

**Authors:** Shi Wang, Zhaoxue Zhang, Rongyu Liu, Shubin Liu, Xiaoyong Liu, Xiuguo Zhang

**Affiliations:** 1 College of Life Sciences, Shandong Normal University, Jinan 250358, China; wangssdau@126.com; 2 Shandong Provincial Key Laboratory for Biology of Vegetable Diseases and Insect Pests, College of Plant Protection, Shandong Agricultural University, Taian 271018, China; zhangzhaoxue2022@126.com (Z.Z.); m2020714642@163.com (R.L.); shubinliu2022@126.com (S.L.)

**Keywords:** *Sordariomycetes*, taxonomy, multigene phylogeny, new taxon

## Abstract

The fungal genus *Gnomoniopsis* (*Gnomoniaceae*, *Diaporthales*) has been reported all around the world and isolated from multiple plant hosts. Based on multilocus phylogenies from a combined dataset of internal transcribed spacer (ITS) region, the ribosomal RNA gene cluster, and partial regions of translation elongation factor 1 alpha (*tef1*) and partial beta-tubulin (*tub2*), in conjunction with morphological characteristics, we describe and illustrate herein four new species, including *Gnomoniopsis* *diaoluoshanensis* sp. Nov., *G. lithocarpi* sp. Nov., *G. mengyinensis* sp. Nov. and *G.* *yunnanensis* sp. Nov. Alongside this, their similarity and dissimilarity to morphologically-allied and phylogenetically-related species are annotated and discussed. For facilitating future identification, we update the key to all species currently recognized in this genus.

## 1. Introduction

*Diaporthales* Nannf. is an important order in the perithecial ascomycetes *Sordariomycetes* Erikss. & Winka, accommodating not only saprophytes but also endophytes or phytopathogens on various hosts [1,2,3,4,5]. *Gnomoniaceae* Winter, which contains 60 genera and 919 species, the second largest family in this order, occurs on growing and overwintering leaves and twigs of hardwood trees, shrubs, and herbaceous plants [6,7]. This family was first established in 1886 [8] and conserved by Hawksworth and Eriksson in 1988 [9,10]. *Gnomoniaceae* was circumscribed by Sogonov et al. in 2008 [11], and since then, their concept has been followed by others. At the present time, besides morphology and molecular data, host specificity has become a key characteristic for species identification and a single species in the *Gnomoniaceae* is often associated with a single host genus or species [6,12,13,14,15,16,17].

*Gnomoniopsis* Berl. was initially described as a subgenus within *Gnomonia* Ces. & De Not. for species with multi-septate ascospores [11]. Subsequently, multiple septa were found not to be a stable characteristic; thus, the *Gnomoniopsis* was synonymized with *Gnomonia* [17]. Currently, *Gnomoniopsis* is accepted as a separate genus in the *Gnomoniaceae* and typified by *Gnomoniopsis chamaemori* (Fr.) Berl. This genus is characterized by having small, black perithecia immersed in the host tissue and one-septate, oval to fusiform ascospores [4]. Species in this genus are delimitated by a combination of morphological and molecular data, and are known to inhabit three plant families only, viz. *Fagaceae*, *Onagraceae* and *Rosaceae* [4,5,11,15,18]. A total of 36 names are documented for *Gnomoniopsis* in the Index Fungorum (accessed on 20 June 2022) and 26 species possess sequence data.

Fungi associated with leaf spots were collected from *Castanea mollissima* Bl. (*Fagaceae*), *Castanopsis chinensis* Hance (*Fagaceae*), and *Lithocarpus fohaiensis* (Hu) A. Camus (*Fagaceae*). We obtained their respective morphological characteristics by separation and purification, using sequences of three molecular markers, including the internal transcribed spacer of ribosomal RNA gene (ITS rDNA), the translation elongation factor 1 alpha gene (*tef1*), and the beta-tubulin gene (*tub2*); we identified these fungi as four species of the genus *Gnomoniopsis*, and proposed them herein.

## 2. Materials and Methods

### 2.1. Isolation and Morphology

Samples of *Castanea mollissima*, *Castanopsis chinensis* and *Lithocarpus fohaiensis* showing necrotic spots were collected from Hainan, Shandong and Yunnan Provinces in China during 2020 and 2021. We obtained a single strain using tissue isolation and single spore isolation. Fragments (5 × 5 mm) were taken from the edges of leaf lesions, surface-sterilized by immersing consecutively in 75% ethanol solution for 1 min and rinsed in sterile distilled water for 30 s, and in 5% sodium hypochlorite solution for 30 s, and then rinsed three times in sterile distilled water for 30 s. The sterilized pieces were placed on sterile filter paper to absorb moisture and then placed on the PDA (PDA: 200 g potato, 20 g dextrose, 20 g agar, 1000 mL distilled water, pH 7.0) and incubated at 25 °C for 2–4 days. Subsequently, portions of agar with fungal mycelia from the periphery of the colonies were transferred onto new PDA plates and photographed on the 7th and 15th days by a digital camera (Canon Powershot G7X).

Micromorphological characters from structures produced in culture were observed using an Olympus SZX10 stereomicroscope and Olympus BX53 microscope, all fitted with an Olympus DP80 high-definition color digital camera to photo-document fungal structures. All fungal strains were stored in 10% sterilized glycerin at 4 ℃ for further studies. Structural measurements were taken using the Digimizer software (https://www.digimizer.com/, accessed on 20 June 2022), with 30 measurements taken for each character [19]. Voucher specimens were deposited in the Herbarium of the Department of Plant Pathology, Shandong Agricultural University, Taian, China (HSAUP) and Herbarium Mycologicum Academiae Sinicae, Institute of Microbiology, Chinese Academy of Sciences, Beijing, China (HMAS). Ex-holotype living cultures were deposited in the Shandong Agricultural University Culture Collection (SAUCC). Taxonomic information of the new taxa was submitted to MycoBank (http://www.mycobank.org, accessed on 20 June 2022).

### 2.2. DNA Extraction and Amplification

Genomic DNA was extracted from mycelia grown on PDA using a CTAB (cetyltrimethylammonium bromide) method [20,21]. Three molecular markers, including an entire internal transcribed spacer region with intervening 5.8S rRNA gene (ITS), partial translation elongation factor 1-alpha gene (*tef1*) and partial beta-tubulin gene (*tub2*), were amplified with the primer pairs and polymerase chain reaction (PCR) programs listed in Table 1. PCR products were separated using the 1% agarose gel with GelRed and UV light was used to visualize the fragments [19]. Sequencing was carried out bidirectionally by the Biosune Company Limited (Shanghai, China). Consensus sequences were obtained using MEGA v. 7.0 [22]. All sequences generated in this study were deposited in GenBank under the accession numbers in Table 2.

### 2.3. Phylogenetic Analyses

The generated sequences for each gene were subjected to BLAST searches for identifying closely related sequences in the NCBI’s GenBank nucleotide database [27]. For the ITS-*tef1*-*tub2* analysis, subsets of sequences from the alignments of Jiang et al. [4] were used as backbones. Newly generated sequences in this study were aligned with additional related sequences downloaded from GenBank (Table 1), using MAFFT 7 online service with the auto strategy (http://mafft.cbrc.jp/alignment/server/, accessed on 20 June 2022) [28]. To establish the identity of the isolates at species level, phylogenetic analyses were conducted first individually for each marker and then combinedly (ITS-*tef1*-*tub2*) (Supplementary File S1).

Phylogenetic analyses were conducted for the multi-marker data based on maximum likelihood (ML) and Bayesian inference (BI) algorithms. For BI, the best evolutionary model for each partition was determined using MrModeltest v. 2.3 [29] and incorporated into the analyses. ML and BI run on the CIPRES Science Gateway portal (https://www.phylo.org/, accessed on 20 June 2022) [30]. ML was performed in RaxML-HPC2 on XSEDE (8.2.12) [31] and 1000 rapid bootstrap replicates were run with the GTRGAMMA model of nucleotide evolution. BI was performed in MrBayes on XSEDE (3.2.7a) [32,33,34]. For ML analyses, the default parameters were used and BI was carried out using the rapid bootstrapping algorithm with the automatic halt option. Bayesian analyses included 4 parallel runs of 5,000,000 generations, with the stop rule option and a sampling frequency of 100 generations. The burn-in fraction was set to 0.25 and posterior probabilities (PP) were determined from the remaining trees. All resulted trees were plotted using FigTree v. 1.4.4 (http://tree.bio.ed.ac.uk/software/figtree, accessed on 20 June 2022) and the layout of the trees was carried out in Adobe Illustrator CC 2019.

## 3. Results

### 3.1. Phylogenetic Analyses

**Figure 1:**
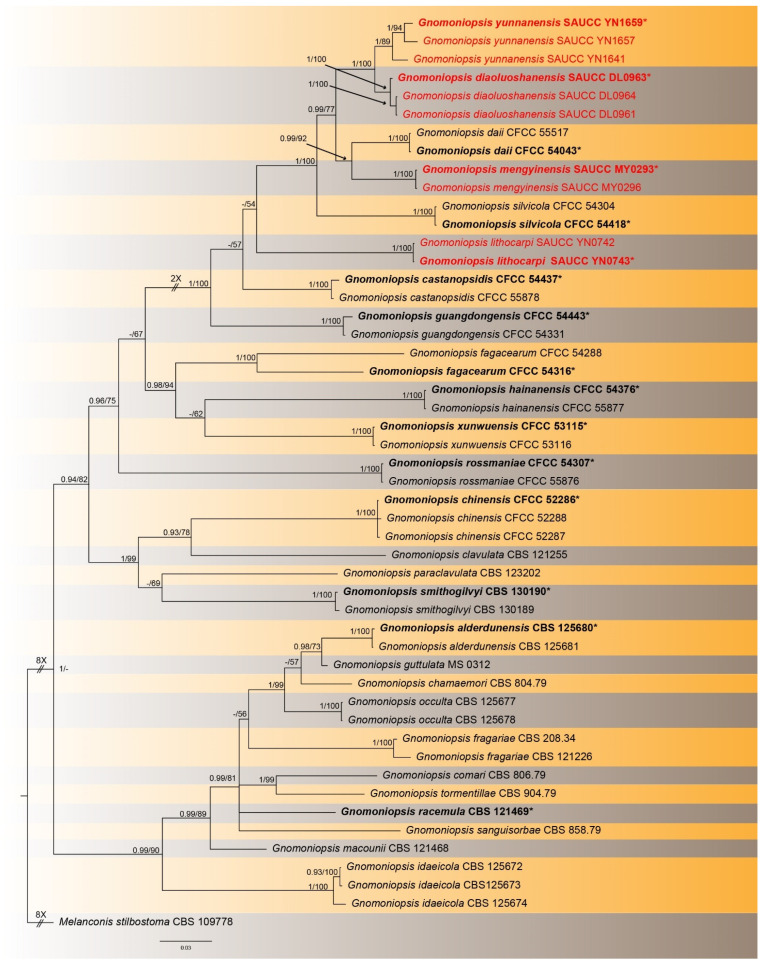
A Bayesian inference phylogram of *Gnomoniopsis* based on combined ITS, *tef1* and *tub2* gene sequences with CBS 109778 of *Melanconis stilbostoma* as the outgroup. At the nodes, the Bayesian inference posterior probability (left, BIPP ≥ 0.90) and the maximum likelihood bootstrap value (right, MLBV ≥ 50%) are separated by a slash. Strains marked with “*” are ex-types or ex-epitypes. Strains from the present study are in red. Some branches are shortened to fit to the page, which are indicated by double slashes and the number of fold times. The scale bar at the bottom middle indicates 0.03 substitutions per site.

The alignment contained 50 isolates representing *Gnomoniopsis* and allied taxa, and the strain CBS 109778 of *Melanconis stilbostoma* was used as the outgroup. A total of 1751 characters were used for phylogenetic analyses, viz. 1–550 (ITS), 551–1222 (*tef1*), 1223–1751 (*tub2*). Of these characters, 979 were constant, 69 were variable and parsimony-uninformative and 703 were parsimony-informative. MrModelTest recommended that the Bayesian inference should use the Dirichlet base frequencies and the GTR+I+G evolutionary mode for all the three partitions. The topology of the Bayesian tree was consistent with that of the ML tree, and therefore is shown as a representative for recapitulating evolutionary history within the genus *Gnomoniopsis* (Figure 1). The final ML optimization likelihood was -13036.518679. The 50 strains were assigned to 28 species clades on the phylogram (Figure 1).

Based on the phylogenetic resolution and morphological analyses, the present study reports four new species of the *Gnomoniopsis* species, viz. *Gnomoniopsis* *diaoluoshanensis* sp. nov., *G. lithocarpi* sp. nov., *G. mengyinensis* sp. nov. and *G. yunnanensis* sp. nov.

### 3.2. Taxonomy

#### 3.2.1. *Gnomoniopsis diaoluoshanensis* S. Wang, Z.X. Zhang, X.Y. Liu and X.G. Zhang, sp. nov.

MycoBank—No: MB844512

Etymology—The epithet *diaoluoshanensis* pertains to the location of the holotype, Diaoluoshan National Silva Park.

Type—China, Hainan Province, Diaoluoshan National Silva Park (18°38′42″–18°50′22″ N, 109°41′38″–110°4′46″ E), on diseased leaves of *Castanopsis chinensis* (*Fagaceae*), 21 May 2021, Z.X. Zhang, holotype HMAS 352166, ex-holotype living culture SAUCC DL0963.

**Figure 2:**
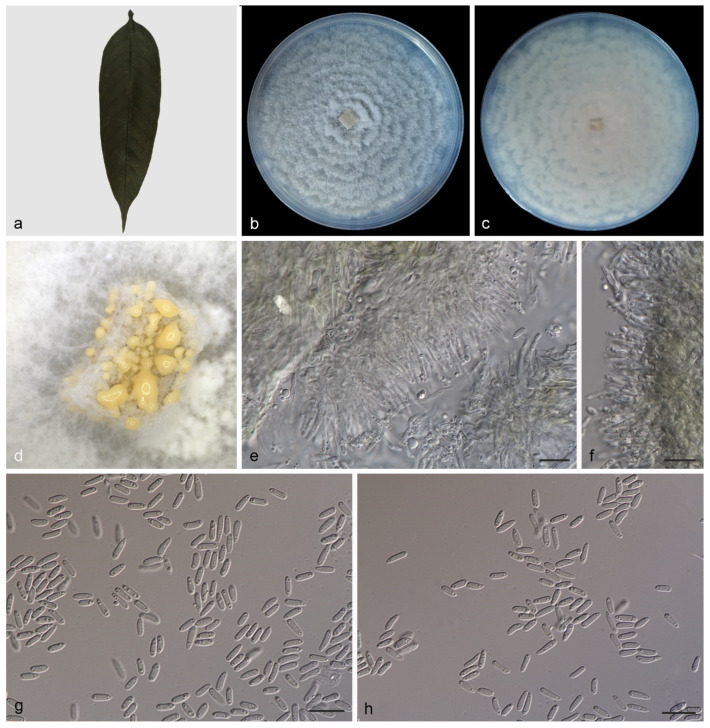
*Gnomoniopsis diaoluoshanensis* (holotype HMAS 352166. (**a**) Leaves of host plant; (**b**,**c**) inverse and reverse sides of colony after 15 days on PDA; (**d**) colony overview; (**e**,**f**) conidiogenous cells and conidia; (**g**,**h**) conidia. Scale bars: (**e**–**h**) 10 μm.

Description—Leaf is endogenic and associated with leaf spots. Conidiomata (pycnothyria) are buried or attached to mycelia, aggregated or solitary, erumpent, exuding creamy yellow conidia after 7 days at 25 ℃ in dark. Conidiophores are indistinct, often reduced. Conidiogenous cells are hyaline, smooth, multi-guttulate, cylindrical to ampulliform, attenuate towards apex, phialidic, 8.0–12.0 × 1.0–2.0 μm. Conidia are hyaline, smooth, multi-guttulate, ellipsoid to broadly ellipsoid, base truncate, 3.8–7.0 × 1.2–2.0 μm, mean = (5.2 ± 0.7) × (1.6 ± 0.2) μm, see Figure 2. Sexual morph was not observed.

Culture characteristics—Colonies on PDA entirely occupy a 90 mm petri dish in 14 days at 25 °C in dark, with a growth rate of 6.0–6.5 mm/day, are grey-white to creamy white with an irregular margin, spreading out in circles in a similar way to petals and the reverse is similar in color.

Additional specimen examined—China, Hainan Province, Diaoluoshan National Silva Park, on diseased leaves of *Castanopsis chinensis* (*Fagaceae*), 21 May 2021, Z.X. Zhang, paratype HMAS 352168, ex-paratype living culture SAUCC DL0961; on diseased leaves of *Castanopsis chinensis* (*Fagaceae*), 21 May 2021, Z.X. Zhang, paratype HMAS 352167, ex-paratype living culture SAUCC DL0964.

Notes—Phylogenetic analyses of a combined three genes (ITS, *tef1* and *tub2*) showed that *Gnomoniopsis diaoluoshanensis* sp. nov. formed an independent clade and is phylogenetically closely related to *G. daii*, *G. mengyinensis* sp. nov. and *G. yunnanensis* sp. nov. (Figure 1). In detail, *G. diaoluoshanensis* is distinguished from *G. daii* by 14/496, 25/314 and 32/445 characters in ITS, *tef1* and *tub2* sequences, respectively. It is distinguished from *G. mengyinensis* by 17/511, 46/638 and 27/467 characters, and from *G. yunnanensis* by 10/508, 28/638 and 6/466. Morphologically, *G. diaoluoshanensis* differs from *G. daii*, *G. mengyinensis* sp. nov. and *G. yunnanensis* sp. nov. mainly in conidia (3.8–7.0 × 1.2–2.0 μm vs. 5.5–7.0 × 2.1–2.5 μm vs. 4.5–6.5 × 1.8–2.8 μm vs. 4.1–5.5 × 1.3–2.0 μm) [4,35].

#### 3.2.2. *Gnomoniopsis lithocarpi* S. Wang, Z.X. Zhang, X.Y. Liu and X.G. Zhang, sp. nov.

MycoBank—No: MB844513

Etymology—The epithet *lithocarpi* pertains to the generic name of the host plant *Lithocarpus fohaiensis*.

Type—China, Yunnan Province, Xishuangbanna Tropical Botanical Garden (21°41′ N, 101°25′ E), Chinese Academy of Sciences, on diseased leaves of *Lithocarpus fohaiensis* (*Fagaceae*), 11 Sep 2020, Z. X. Zhang, holotype HMAS 352165, ex-holotype living culture SAUCC200743.

**Figure 3:**
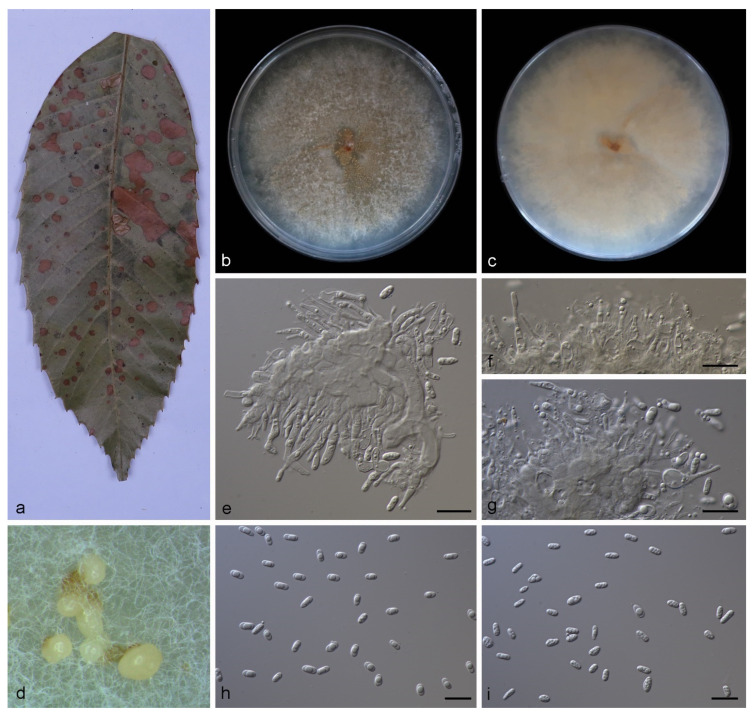
*Gnomoniopsis lithocarpi* (holotype HMAS 352165). (**a**) Leaves of host plant; (**b**,**c**) inverse and reverse sides of colony after 15 days on PDA; (**d**) colony overview; (**e**–**g**) conidiogenous cells and conidia; (**h**,**i**) conidia. Scale bars: (**e**–**i**) 10 μm.

Description—Leaf is endogenic and associated with leaf spots. Conidiomata (pycnothyria) are buried or attached to mycelia, aggregated or solitary, erumpent, exuding pale yellow conidia after 14 days at 25 °C in dark. Conidiophores are indistinct, often reduced. Conidiogenous cells are hyaline, smooth, multi-guttulate, cylindrical to ampulliform, attenuate towards apex, phialidic, 6.0–13.0 × 1.5–2.5 μm. Conidia are hyaline, smooth, multi-guttulate, ellipsoid to ovoid, base circular, 4.0–5.8 × 1.7–2.4 μm, mean = (4.6 ± 0.5) × (2.1 ± 0.2) μm, see Figure 3. Sexual morph was not observed.

Culture characteristics—Colonies on PDA at 25 °C for 14 days in dark reach 75–80 mm in diameter, are circular, with moderate aerial mycelia on the surface, light brown and sparse in the center, white and dense at the edge and the reverse is similar in color.

Additional specimen examined—China, Yunnan Province, Xishuangbanna Tropical Botanical Garden, Chinese Academy of Sciences on diseased leaves of *Lithocarpus fohaiensis* (*Fagaceae*), 11 Sep 2020, Z.X. Zhang, paratype HMAS 352164, ex-paratype living culture SAUCC YN0742.

Notes—Phylogenetic analyses of three combined genes (ITS, *tef1* and *tub2*) showed that *Gnomoniopsis lithocarpi* formed an independent clade closely related to *G. castanopsidis* and *G. silvicola*. The *G.* *lithocarpi* sp. nov. is distinguished from *G. castanopsidis* by 35/513, 41/325 and 48/478 characters in ITS, *tef1* and *tub2* sequences, respectively, and from *G. silvicola* by 38/517, 42/325 and 58/470 characters. Morphologically, *G.* *lithocarpi* differs from *G. castanopsidis* and *G. silvicola* in conidia (4.0–5.8 × 1.7–2.4 μm vs. 4.5–5.3 × 2.2–2.7 μm vs. 4.6–5.1 × 2.1–2.5 μm), and in colony texture (light brown to white on PDA and dense at the edge vs. dirty-white to fawn on PDA and undulate margin vs. dirty-white on PDA and undulate margin) [4,35].

#### 3.2.3. *Gnomoniopsis* *mengyinensis* S. Wang, Z.X. Zhang, X.Y. Liu and X.G. Zhang, sp. nov.

MycoBank No.: MB844514

Etymology—The epithet *mengyinensis* pertains to the location where the holotype was collected, Mengyin County.

Type—China, Shandong Province, Mengyin County (35°71′ N, 117°94′ E), on diseased leaves of *Castanea mollissima* (*Fagaceae*), 25 July 2020, Z.X. Zhang, holotype HMAS 352160, ex-holotype living culture SAUCC MY0293.

**Figure 4:**
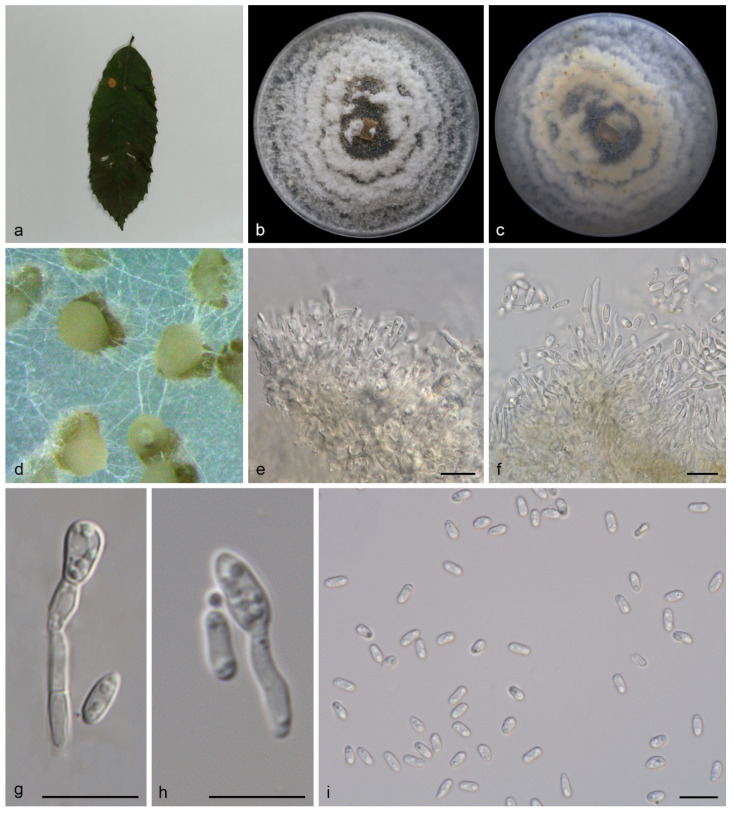
*Gnomoniopsis mengyinensis* (holotype HMAS 352160). (**a**) Leaves of host plant; (**b**,**c**) inverse and reverse sides of colony after 14 days on PDA; (**d**) colony overview; (**e**–**h**) conidiogenous cells and conidia; (**i**) conidia. Scale bars: (**e**–**i**) 10 μm.

Description—Leaf is endogenic and associated with leaf spots. Conidiomata (pycnothyria) are aggregated or solitary, erumpent, globose to pulvinate, light brown, exuding creamy white or hyaline conidial after 10 days at 25 °C in dark. Conidiophores are indistinct, often reduced. Conidiogenous cells are hyaline, cylindrical, attenuate towards apex, phialidic, 8.0–11.5 × 1.3–2.2 μm. Conidia are hyaline, smooth, multi-guttulate, cylindrical, oval to fusoid, straight or slightly curved, truncate at the base, 4.5–6.5 × 1.8–2.8 μm, mean = (5.4 ± 0.4) × (2.2 ± 0.2) μm, see Figure 4. Sexual morph is unknown.

Culture characteristics—Cultures incubated on PDA at 25 °C in dark attain 82.0–86.0 mm in diameter after 14 days, with a growth rate of 5.8–6.2 mm diam/day and the colonies are flat, spreading with moderate aerial mycelia and lobate to undulate margins, grey-white to creamy, spreading out in a similar way to petals and the reverse is similar in color.

Additional specimen examined—China, Shandong Province, Mengyin County, on diseased leaves of *Castanea mollissima* (*Fagaceae*), 25 July 2020, Z.X. Zhang, paratype HMAS 352159, ex-prartype living culture SAUCC MY0296.

Notes—In the phylogenetic tree (Figure 1), *Gnomoniopsis mengyinensis* sp. nov. is closely related to *G. daii* (BIPP = 0.97, MLBS = 95%). This new species is distinguished from *G. daii* by a total of 65 characters in the concatenated sequence alignment (5/509 in the ITS, 29/313 in the *tef1* and 22/442 in the *tub2*). Morphologically, *Gnomoniopsis mengyinensis* differs from *G. daii* in conidia (4.5–6.5 × 1.8–2.8 μm vs. 5.1–6.3 × 2.8–3.2 μm), conidiogenous cells (4.5–6.5 × 1.8–2.8 μm vs. 5.6–6.1 × 2.8–3.2 μm), as well as conidiomatum color (light brown vs. dark brown) [4,35].

#### 3.2.4. *Gnomoniopsis yunnanensis* S. Wang, Z.X. Zhang, X.Y. Liu and X.G. Zhang, sp. nov.

MycoBank—No: MB844515

Etymology—The epithet *yunnanensis* pertains to the location where the holotype was collected, Yunnan Province.

Type—China, Yunnan Province, Xishuangbanna Tropical Botanical Garden (21°41′N, 101°25′E), Chinese Academy of Sciences, on diseased leaves of *Castanea mollissima* (*Fagaceae*), 11 Sep 2020, Z. X. Zhang, holotype HMAS 352161, ex-holotype living culture SAUCC YN1659.

**Figure 5:**
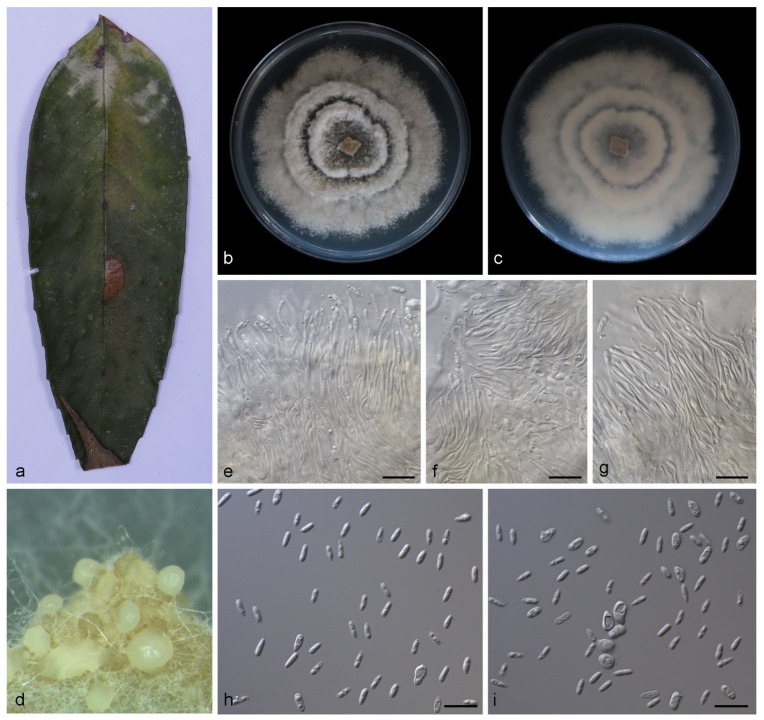
*Gnomoniopsis yunnanensis* (holotype HMAS 352161). (**a**) Leaves of host plant; (**b**,**c**) inverse and reverse sides of colony after 15 days on PDA; (**d**) colony overview; (**e**–**g**) conidiogenous cells and conidia; (**h**,**i**) conidia. Scale bars: (**e**–**i**) 10 μm.

Description—Leaf is endogenic and associated with leaf spots. Conidiomata (pycnothyria) are aggregated or solitary, erumpent, globose to pulvinate, light yellow, exuding creamy white or hyaline conidia after 14 days at 25 °C in dark. Conidiophores are indistinct, often reduced. Conidiogenous cells are hyaline, cylindrical, attenuate towards apex, phialidic, 9.0–18.0 × 0.5–1.57 μm. Conidia are hyaline, smooth, multi-guttulate, cylindrical, oblong to ellipsoid, straight or slightly curved, truncate at the base, 4.1–5.5 × 1.3–2.0 μm, mean = (4.9 ± 0.4) × (1.6 ± 0.2) μm, see Figure 5. Sexual morph is unknown.

Culture characteristics—Cultures incubated on PDA at 25 °C for 14 days in dark attain 69.0–72.0 mm in diameter, with a growth rate of 4.9–5.2 mm diam/day, with moderate aerial mycelia and a lobate to undulate margin, grey-white to creamy, spreading out in a similar way to petals and the reverse is similar in color.

Additional specimen examined—China, Yunnan Province, Xishuangbanna Tropical Botanical Garden, Chinese Academy of Sciences, on diseased leaves of *Castanea mollissima* (*Fagaceae*), 11 Sep 2020, Z.X. Zhang, paratype HMAS 352162, ex-paratype living culture SAUCC YN1657; on diseased leaves of *Castanea mollissima* (*Fagaceae*), 11 Sep 2020, Z.X. Zhang, paratype HMAS 352163, ex-paratype living culture SAUCC YN1641.

Notes—Strains SAUCC YN1659, SAUCC YN1657 and SAUCC YN1641 are identified to the same species *Gnomoniopsis yunnanensis* sp. nov. on the basis of similar morphology and molecular monophyly. For details, one can refer to the notes for *G. diaoluoshanensis*.

### 3.3. Key to the Species of Gnomoniopsis

Together with the 4 new species proposed in this study, we have currently accepted a worldwide total of 30 species in the genus *Gnomoniopsis*. In order to facilitate identification in the future, a key to the species of *Gnomoniopsis* is provided herein. Characteristics adopted in the key include perithecia, septa, asci, ascospores, conidiogenous cells, conidia, and chlamydospores.

1. Sexual morph known------------------------------------------------------------------------------------21. Sexual morph unknown-------------------------------------------------------------------------------162. Asci cylindrical--------------------------------------------------------------------------------------------32. Asci fusiform-----------------------------------------------------------------------------------------------43. Ascospores size 10.0–13.0 × 2.0–3.0 μm---------------------------------------------*G. chamaemori*3. Ascospores size 4.0–12.0 × 1.0–3.0 μm----------------------------------------------*G. smithogilvyi*4. Perithecia without stroma-------------------------------------------------------------------------------54. Perithecia with stroma-----------------------------------------------------------------------------------75. Perithecia immersed--------------------------------------------------------------------*G. sanguisorbae*5. Perithecia superficies-------------------------------------------------------------------------------------66. Perithecia size 110.0–150.0 × 120.0–140.0 μm-----------------------------------*G. clavulata*6. Perithecia size 139.0–180.0 × 156.0–241.0 μm-----------------------------*G. paraclavulata*7. Ascospores aseptate--------------------------------------------------------------------------------------87. Ascospores septate--------------------------------------------------------------------------------------108. Perithecia groups----------------------------------------------------------------------------*G. racemula*8. Perithecia solitary-----------------------------------------------------------------------------------------99. Ascospores size 6.0–10.0 × 1.5–3.0 μm----------------------------------------------*G. tormentillae*9. Ascospores size 7.0–8.0 × 1.8–2.2 μm-------------------------------------------------*G. agrimoniae*10. Perithecia surfaced on the host---------------------------------------------------------------------1110. Perithecia immersed in the host--------------------------------------------------------------------1211. Perithecia size 280.0–375.0 × 327.0–490.0 μm----------------------------------*G. alderdunensis*11. Perithecia size 112–330.0 × 125–500.0 μm-----------------------------------------------*G. comari*12. Perithecia immersed in stem------------------------------------------------------------------------1312. Perithecia immersed in leaves----------------------------------------------------------------------1413. Asci size 30.0–48.5 × 5.0–10.0-------------------------------------------------------------*G. idaeicola*13. Asci size 30.0–38.0 × 4.0–8.5--------------------------------------------------------------*G. macounii*14. Perithecia aggregated 2–4----------------------------------------------------------------*G. guttulata*14. Perithecia solitary--------------------------------------------------------------------------------------1515. Perithecia size 150.0–475.0 × 200.0–475.0 μm----------------------------------------*G. fragariae*15. Perithecia size 129.0–340.0 × 147.0–428.0 μm-------------------------------------------*G. occulta*16. Conidiogenous cells guttulate----------------------------------------------------------------------1716. Conidiogenous cells no guttulate------------------------------------------------------------------2417. Conidia base circular--------------------------------------------------------***G. lithocarpi sp. nov.***
17. Conidia base truncate---------------------------------------------------------------------------------1818. Conidia ellipsoid or cylindrical---------------------------------------------------------------------1918. Conidia oval or fusoid--------------------------------------------------------------------------------2019. Conidiogenous cells size 8.0–12.0 × 1.0–2.0 μm-------------***G. diaoluoshanensis sp. nov.***
19. Conidiogenous cells size 12.5–24.0 × 1.5–3.0 μm---------------------------*G. guangdongensis*20. Conidia 1-septate-------------------------------------------------------------------------*G. rossmaniae*20. Conidia aseptate----------------------------------------------------------------------------------------2121. Conidia maximum length < 6.0 μm----------------------------------------------------------------2221. Conidia maximum length > 6.0 μm----------------------------------------------------------------2322. Conidiogenous cells 6.5–13.0 × 1.5–3.0 μm--------------------------------------*G. castanopsidis*22. Conidiogenous cells 7.0–15.0 × 1.5–2.5 μm--------------------------------------------*G. silvicola*23. Conidiogenous cells 16.0–33.5 × 2.0–5.0--------------------------------------------*G. fagacearum*23. Conidiogenous cells 16.5–26.0 × 2.5–4.5-------------------------------------------*G. hainanensis*24. Conidiogenous cells one-celled---------------------------------------------------------------------2524. Conidiogenous cells multi-celled------------------------------------------------------------------2625. Conidia 1-septate---------------------------------------------------------------------------*G. chinensis*25. Conidia aseptate-----------------------------------------------------------------------------------*G. daii*26. Conidiogenous cells branched-------------------------------------------------------*G. xunwuensis*26. Conidiogenous cells unbranched------------------------------------------------------------------2727. Conidia maximum length < 10.0 μm--------------------------------------------------------------2827. Conidia maximum length > 10.0 μm--------------------------------------------------------------2928. Conidia oval to fusoid--------------------------------------------------***G. mengyinensis sp. nov.***
28. Conidia oblong to ellipsoid---------------------------------------------***G. yunnanensis sp. nov.***
29. Conidia subcylindrical------------------------------------------------------------------*G. angolensis*29. Conidia fusoid-----------------------------------------------------------------------------------*G. rosae*

## 4. Discussion

In the present study, four new species (*Gnomoniopsis* *diaoluoshanensis*, *G. lithocarpi*, *G. mengyinensis*, and *G. yunnanensis*) from three hosts (*Castanea mollissima*, *Castanopsis chinensis*, and *Lithocarpus fohaiensis*) in three provinces of China were described and illustrated (Figure 2, Figure 3, Figure 4 and Figure 5), and all these three hosts belong to the family *Fagaceae*. Currently, *Gnomoniopsis* species were found from hosts that belong to three plant families (*Fagaceae*, *Onagraceae* and *Rosaceae*). Sixteen *Gnomoniopsis* species (including the four new species herein) were described from fagaceous hosts. Only one species (*G. racemula*) was described from the *Onagraceae* family [11,15,36]. The remaining 11 species were from the family *Rosaceae*. The *Fagaceae*, *Onagraceae* and *Rosaceae* plants are widely distributed in China, suggesting abundant potentially new *Gnomoniopsis* species.

Driven by recent developments in DNA sequence analyses, taxonomists have combined phylogenetic data to gain insights into evolutionary relationships [37,38,39]. Jiang et al. [4] introduced six species in *Gnomoniopsis*, based on three gene loci encoding the internal transcribed spacer of ribosomal RNA (ITS), translation elongation factor 1 alpha (*tef1*), and beta-tubulin (*tub2*). They described and illustrated the *Gnomoniopsis* species from seven regions (Fujian, Guangdong, Hainan, Henan, Jiangxi and Shaanxi) in China. In sum, 13 species of *Gnomoniopsis* were recorded in more than 10 regions of China, and they are *Gnomoniopsis castanopsidis*, *G. chinensis*, *G. daii*, *G. diaoluoshanensis*, *G. fagacearum*, *G. guangdongensis*, *G. hainanensis*, *G. lithocarpi*, *G. mengyinensis*, *G. rossmaniae*, *G. silvicola*, *G. xunwuensis* and *G. yunnanensis*.

The *Gnomoniopsis* species were reported with 200 records in Fungal Databases (https://nt.ars-grin.gov/fungaldatabases/index.cfm, accessed on 20 June 2022). Among these, *G. daii* and *G. chinensis* were determined to be phytopathogenic, causing fruit rot and leaf spot diseases and branch canker of Chinese chestnut, respectively [40,41]. *Gnomoniopsis smithogilvyi* were illustrated and described in 12 countries (Australia, New Zealand, Chile, France, India, Ireland, Italy, Portugal, Spain, Switzerland, United Kingdom and USA) with 30 records in Fungal Databases, causing sweet chestnut branch canker and fruit rot in Australia, Europe and USA [42,43,44]. Apart from this, Linaldeddu et. al. revealed some fungi associated with branch diseases of hazelnut in Sardinia (Italy), including *Dothiorella iberica*, *Do. omnivora*, *Do. symphoricarposicola* and *G. smithogilvyi*. *Gnomoniopsis smithogilvyi* was isolated from rotting chestnut kernels as an endophyte from asymptomatic flowers, leaves and stems of the genus Chestnut [45]. The descriptions, pathogenicity testing and molecular data for species of *Gnomoniopsis* by taxonomists represent an important resource for plant pathologists and plant quarantine officials.

## Figures and Tables

**Table 1:** Molecular markers and their PCR primers and programs used in this study.

Loci	PCR Primers	Sequence (5′—3′)	PCR Cycles	References
ITS	ITS5 ITS4	GGA AGT AAA AGT CGT AAC AAG G TCC TCC GCT TAT TGA TAT GC	(95 °C: 30 s, 55 °C: 30 s, 72 °C: 1 min) × 35 cycles	[23]
*tef1*	EF1-728F EF-2	CAT CGA GAA GTT CGA GAA GG GGA RGT ACC AGT SAT CAT GTT	(95 °C: 30 s, 48 °C: 30 s, 72 °C: 1 min) × 35 cycles	[24,25]
*tub2*	Bt-2a Bt-2b	GGT AAC CAA ATC GGT GCT GCT TTC ACC CTC AGT GTA GTG ACC CTT GGC	(95 °C: 30 s, 53 °C: 30 s, 72 °C: 1 min) × 35 cycles	[26]

**Table 2:** Information of specimens used in this study.

Species	Voucher	Host	Country	GenBank Accession Number		
				ITS	*tef1*	*tub2*
*Gnomoniopsis alderdunensis*	CBS 125680 *	*Rubus parviflorus* (*Rosaeace*)	USA	GU320825	GU320801	GU320787
	CBS 125681	*Rubus parviflorus* (*Rosaeace*)	USA	GU320827	GU320802	GU320789
*G. castanopsidis*	CFCC 54437 *	*Castanopsis hystrix* (*Fagaceae*)	China	MZ902909	MZ936385	–
	CFCC 54438	*Castanopsis hystrix* (*Fagaceae*)	China	MZ902910	MZ936386	–
*G. chamaemori*	CBS 804.79	*Rubus chamaemorus* (*Rosaeace*)	Finland	GU320817	GU320809	GU320777
*G. chinensis*	CFCC 52286 *	*Castanea mollissima* (*Fagaceae*)	China	MG866032	MH545370	MH545366
	CFCC 52288	*Castanea mollissima* (*Fagaceae*)	China	MG866034	MH545372	MH545368
	CFCC 52287	*Castanea mollissima* (*Fagaceae*)	China	MG866033	MH545371	MH545367
*G. clavulata*	CBS 121255	*Quercus falcata* (*Fagaceae*)	USA	EU254818	EU221934	EU219211
*G. comari*	CBS 806.79	*Oryza sativa* (*Rosaeace*)	UK	EU254821	GU320810	EU219156
*G. daii*	CFCC 54043 *	*Castanea mollissima* (*Fagaceae*)	China	MZ902911	MZ936387	MZ936403
	CFCC 55517	*Castanea mollissima* (*Fagaceae*)	China	MN598671	MN605517	MN605519
***G. diaoluoshanensis***	SAUCC DL0963 *	***Castanopsis chinensis*** **(*Fagaceae*)**	**China**	**ON753744**	**ON759769**	**ON759777**
	**SAUCC DL0964**	***Castanopsis chinensis*** **(*Fagaceae*)**	**China**	**ON753743**	**ON759768**	**ON759776**
	**SAUCC DL0961**	***Castanopsis chinensis*** **(*Fagaceae*)**	**China**	**ON753745**	**ON759770**	**ON759778**
*G. fagacearum*	CFCC 54316 *	*Lithocarpus glaber* (*Fagaceae*)	China	MZ902916	MZ936392	MZ936408
	CFCC 54288	*Castanopsis faberi* (*Fagaceae*)	China	MZ902913	MZ936389	MZ936405
*G. fragariae = G. fructicola*	CBS 208.34	*Fragaria* sp. (*Rosaeace*)	USA	EU254826	EU221968	EU219149
	CBS 121226	*Fragaria vesca* (*Rosaeace*)	USA	EU254824	EU221961	EU219144
*G. guangdongensis*	CFCC 54443 *	*Castanopsis fargesii* (*Fagaceae*)	China	MZ902918	MZ936394	MZ936410
	CFCC 54331	*Castanopsis fargesii* (*Fagaceae*)	China	MZ902919	MZ936395	MZ936411
*G. guttulata*	MS 0312	*Agrimonia eupatoria* (*Rosaeace*)	Bulgaria	EU254812	–	–
*G. hainanensis*	CFCC 54376 *	*Castanopsis hainanensis* (*Fagaceae*)	China	MZ902921	MZ936397	MZ936413
	CFCC 55877	*Castanopsis hainanensis* (*Fagaceae*)	China	MZ902922	MZ936398	MZ936414
*G. idaeicola*	CBS 125672	*Rubus* sp. (*Rosaeace*)	USA	GU320823	GU320797	GU320781
	CBS 125673	*Rubus pedatus* (*Rosaeace*)	USA	GU320824	GU320798	GU320782
	CBS 125674	*Rubus* sp. (*Rosaeace*)	France	GU320820	GU320796	GU320780
***G. lithocarpi***	SAUCC YN0743 *	***Lithocarpus fohaiensis* (*Fagaceae*)**	**China**	**ON753749**	**ON759765**	**ON759783**
	**SAUCC YN0742**	***Lithocarpus fohaiensis* (*Fagaceae*)**	**China**	**ON753750**	**ON759764**	**ON759782**
*G. macounii*	CBS 121468	*Spiraea* sp. (*Rosaeace*)	USA	EU254762	EU221979	EU219126
***G. mengyinensis***	SAUCC MY0293 *	***Castanea mollissima* (*Fagaceae*)**	**China**	**ON753741**	**ON759766**	**ON759774**
	**SAUCC MY0296**	***Castanea mollissima* (*Fagaceae*)**	**China**	**ON753742**	**ON759767**	**ON759775**
*G. occulta*	CBS 125677	*Potentilla* sp. (*Rosaeace*)	USA	GU320828	GU320812	GU320785
	CBS 125678	*Potentilla* sp. (*Rosaeace*)	USA	GU320829	GU320800	GU320786
*G. paraclavulata*	CBS 123202	*Agrostis* sp. (*Fagaceae*)	USA	GU320830	GU320815	GU320775
*G. racemula*	CBS 121469 *	*Triticum aestivum* (*Onagraceae*)	USA	EU254841	EU221889	EU219125
*G. rossmaniae*	CFCC 54307 *	*Castanopsis hainanensis* (*Fagaceae*)	China	MZ902923	MZ936399	MZ936415
	CFCC 55876	*Castanopsis hainanensis* (*Fagaceae*)	China	MZ902924	MZ936400	MZ936416
*G. sanguisorbae*	CBS 858.79	*Sanguisorba minor* (*Rosaeace*)	Switzerland	GU320818	GU320805	GU320790
*G. silvicola*	CFCC 54304	*Castanopsis hystrix* (*Fagaceae*)	China	MZ902925	MZ936401	MZ936417
	CFCC 54418 *	*Quercus serrata* (*Fagaceae*)	China	MZ902926	MZ936402	MZ936418
*G. smithogilvyi*	CBS 130190 *	*Castanea* sp. (*Fagaceae*)	Australia	JQ910642	JQ910645	JQ910639
	CBS 130189	*Castanea* sp. (*Fagaceae*)	Australia	JQ910644	JQ910647	JQ910641
*G. tormentillae*	CBS 904.79	*Potentilla* sp. (*Rosaeace*)	Switzerland	EU254856	GU320795	EU219165
*G. xunwuensis*	CFCC 53115 *	*Castanopsis fissa* (*Fagaceae*)	China	MK432667	MK578141	MK578067
	CFCC 53116	*Castanopsis fissa* (*Fagaceae*)	China	MK432668	MK578142	MK578068
***G.*** ***yunnanensis***	SAUCC YN1659 *	***Castanea mollissima* (*Fagaceae*)**	**China**	**ON753746**	**ON759771**	**ON759779**
	**SAUCC YN1657**	***Castanea mollissima* (*Fagaceae*)**	**China**	**ON753747**	**ON759772**	**ON759780**
	**SAUCC YN1641**	***Castanea mollissima* (*Fagaceae*)**	**China**	**ON753748**	**ON759773**	**ON759781**
*Melanconis stilbostoma*	CBS 109778	*Betula pendula* (*Betulaceae*)	Australia	DQ323524	EU221886	EU219104

Notes: New species established in this study are in bold. Ex-type or ex-epitype strains are marked with “*”.

## Data Availability

The sequences from the present study were submitted to the NCBI database (https://www.ncbi.nlm.nih.gov/, accessed on 20 June 2022) and the accession numbers were listed in Table 2.

## Supplementary Materials

The following supporting information can be downloaded at: https://www.mdpi.com/article/10.3390/jof8080770/s1, Supplementary File S1: The combined ITS, tef1 and tub2 sequences.
